# 87. Impact of Universal Decolonization With and Without Enhanced Cleaning on MultiDrug-Resistant Organism (MDRO) Body-Site Carriage and Environmental Contamination in Nursing Homes (NHs): The CLEAN Study

**DOI:** 10.1093/ofid/ofac492.012

**Published:** 2022-12-15

**Authors:** Gabrielle M Gussin, Raveena D Singh, Thomas T Tjoa, Chase Berman, Raheeb Saavedra, Jessica Macapulay, Robert Pedroza, Karina Gordeychev, Julie A Shimabukuro, Kaye D Evans, Cassiana E Bittencourt, Susan S Huang

**Affiliations:** University of California, Irvine School of Medicine, Division of Infectious Diseases, Irvine, California; University of California, Irvine School of Medicine, Division of Infectious Diseases, Irvine, California; University of California, Irvine School of Medicine, Division of Infectious Diseases, Irvine, California; University of California, Irvine School of Medicine, Division of Infectious Diseases, Irvine, California; University of California, Irvine School of Medicine, Division of Infectious Diseases, Irvine, California; University of California, Irvine School of Medicine, Division of Infectious Diseases, Irvine, California; University of California, Irvine School of Medicine, Division of Infectious Diseases, Irvine, California; University of California, Irvine School of Medicine, Division of Infectious Diseases, Irvine, California; University of California, Irvine Health, Orange, California; University of California, Irvine Health, Orange, California; University of California, Irvine Health, Orange, California; University of California, Irvine School of Medicine, Irvine, CA

## Abstract

**Background:**

Decolonization (decol) and environmental cleaning are strategies to reduce MDRO transmission and infection. While decol in NHs has been proven to reduce MDRO carriage, infection-related hospitalization, and contamination, the impact of enhanced cleaning (EC) with or without decol has not been evaluated.

**Methods:**

We performed a 4-phase study in 2 NHs from 2019–21 to compare 1) decol, 2) EC, and 3) decol plus EC on MDRO carriage and contamination vs routine care (control). Decol involved nasal iodophor on admission x 5d and then M-F biweekly plus chlorhexidine for routine bathing for all residents. EC involved standardized training for cart setup, disinfectant preparation and saturation, color-coded microfiber cloths for specific items, and UV marker feedback. Outcomes were serial MDRO point prevalence (6 per phase) of nares/skin and MDRO contamination of high-touch bedroom and common area objects. Swabs were processed for MRSA, VRE, ESBL, and CRE. We used generalized linear mixed models to compare phases while clustering by NH. Analyses of objects also clustered by room.

**Results:**

Table 1 shows crude MDRO prevalence and environmental contamination by study phase. Table 2 shows the impact of each phase vs control. Decol had the greatest impact on MDRO carriage with a 46% reduction vs control. EC alone did not decrease MDRO carriage, and EC plus decol did not further reduce MDRO carriage vs decol alone. The impact of decol vs EC on carriage differed for Gram-positive (GP) vs negative (GN) pathogens. Only decol reduced MRSA and VRE carriage, while both decol and EC reduced GN carriage. The reduction in GN carriage was enhanced with both decol and EC, although this was not statistically significant. With EC, 24-hour UV marker removal of high-touch objects increased from 7% to 45%. Decol and EC both reduced MDRO object contamination, by 84% and 75%, respectively. The impact of decol on object contamination was not enhanced with EC.

This table shows the crude MDRO prevalence and environmental contamination results by study phase. Each phase involved 6 serial point prevalence sweeps of 40 residents per nursing home (N=480 total residents per phase), and 6 serial sweeps of high-touch bedroom and common area objects (N=504 total objects per phase).

This table shows the odds ratios and 95% confidence intervals for any MDRO and specific MDRO-types for each study phase for the outcomes of 1) MDRO body-site prevalence and 2) MDRO contamination of high-touch objects. The P-value reported represents the overall comparison of the intervention study phases versus control (routine care).

**Conclusion:**

Multimodal strategies are needed to reduce MDRO burden in NHs. While both decolonization and cleaning independently reduced MDRO object contamination, only decolonization significantly reduced MDRO carriage prevalence. Decolonization provided substantial reductions in MDRO carriage and environmental contamination compared to cleaning alone.

**Disclosures:**

**Gabrielle M. Gussin, MS**, Medline: Conducted studies in which hospitals and nursing homes received contributed antiseptic and/or environmental cleaning products|Stryker: Conducted clinical studies in which hospitals and nursing homes received contributed antiseptic products|Xttrium Laboratories: Conducted clinical studies in which hospitals and nursing homes received contributed antiseptic products **Raveena D. Singh, MA**, Medline: Conducted studies in which hospitals and nursing homes received contributed antiseptic and/or environmental cleaning products|Stryker: Conducted clinical studies in which hospitals and nursing homes received contributed antiseptic products|Xttrium Laboratories: Conducted clinical studies in which hospitals and nursing homes received contributed antiseptic products **Raheeb Saavedra, AS**, Medline: Conducted studies in which hospitals and nursing homes received contributed antiseptic and/or environmental cleaning products|Stryker: Conducted clinical studies in which hospitals and nursing homes received contributed antiseptic products|Xttrium Laboratories: Conducted clinical studies in which hospitals and nursing homes received contributed antiseptic products **Susan S. Huang, MD, MPH**, Medline: Conducted studies in which hospitals and nursing homes received contributed antiseptic and/or environmental cleaning products|Molnlyke: Conducted clinical studies in which hospitals received contributed antiseptic product|Stryker: Conducted clinical studies in which hospitals and nursing homes received contributed antiseptic products|Xttrium Laboratories: Conducted clinical studies in which hospitals and nursing homes received contributed antiseptic product.

